# Supplementary material for: Chromothripsis during telomere crisis is independent of NHEJ, and consistent with a replicative origin
Source: Genome Res. 2019 May;29(5):737–49. doi: 10.1101/gr.240705.118 (PMC6499312; doi:10.1101/gr.240705.118)
Supplement: Supplemental Material [file supp_gr.240705.118_Supplemental_file_1.zip › contigs/annotated_contigs/DB79/contig.2.DB79_length_283_mean_cov_8.57597173145.docx]

**DB79_length_283_mean_cov_8.57597173145**

```
GATTCTTCTGCCTCAGCCTCCCGAGTAGCTGAGATTACAGGTGTCGCGCCACCATGCCCGGCTAGTTTTTTGTATTTTTAGTAGAGATG
 >chr17:76843670-76843862 + E=6e-90
CGATTTCACCATGTTGGCCAGGCTGGTCTTGAACCCCTGACCTCAGGTGATCCACCCTCCTTGGCCTCCCAAAGTGCTGGGATTACAGG

CGTGAGCCACCGCGCCT|ATATATATACTATATATATACAAATCTCTT|AGGGGTTCTAAGAGATTTGAGCCGACCACGTGGGGTGGGG
                                                >chr17:76871792-76871850 - E=1e-23
CTGGGGCTGGGGCTAGGA
```
